# Deficits in working memory, reading comprehension and arithmetic skills in children with mouth breathing syndrome: analytical cross-sectional study: Déficits de memória operacional, compreensão de leitura e habilidades aritméticas em crianças com síndrome da respiração oral: estudo transversal analítico

**DOI:** 10.1590/1516-3180.2013.7630011

**Published:** 2014-09-26

**Authors:** Rita Cristina Sadako Kuroishi, Ricardo Basso Garcia, Fabiana Cardoso Pereira Valera, Wilma Terezinha Anselmo-Lima, Marisa Tomoe Hebihara Fukuda

**Affiliations:** I BSc, MSc. Speech Therapist, Department of Ophthalmology, Otolaryngology and Head and Neck Surgery, Faculdade de Medicina de Ribeirão Preto (FMRP), Universidade de São Paulo (USP), Ribeirão Preto, São Paulo, Brazil.; II BA, MA, PhD. Collaborator in the Department of Psychology, Faculdade de Filosofia, Ciências e Letras de Ribeirão Preto (FFCLRP), Universidade de São Paulo (USP), Ribeirão Preto, São Paulo, Brazil.; III MD, MSc, PhD. Professor, Department of Ophthalmology, Otolaryngology and Head and Neck Surgery, Faculdade de Medicina de Ribeirão Preto (FMRP), Universidade de São Paulo (USP), Ribeirão Preto, São Paulo, Brazil.; IV MD, MSc, PhD. Full Professor, Department of Ophthalmology, Otolaryngology and Head and Neck Surgery, Faculdade de Medicina de Ribeirão Preto (FMRP), Universidade de São Paulo (USP), Ribeirão Preto, São Paulo, Brazil.; V BSc, MSc, PhD. Professor, Department of Ophthalmology, Otolaryngology and Head and Neck Surgery, Faculdade de Medicina de Ribeirão Preto (FMRP), Universidade de São Paulo (USP), Ribeirão Preto, São Paulo, Brazil.

**Keywords:** Mouth breathing, Memory, short-term, Cognition, Underachievement, Public health

## Abstract

**CONTEXT AND OBJECTIVE:**

Mouth breathing syndrome is very common among school-age children, and it is possibly related to learning difficulties and low academic achievement. In this study, we investigated working memory, reading comprehension and arithmetic skills in children with nasal and mouth breathing.

**DESIGN AND SETTING:**

Analytical cross-sectional study with control group conducted in a public university hospital.

**METHODS:**

42 children (mean age = 8.7 years) who had been identified as mouth breathers were compared with a control group (mean age = 8.4 years) matched for age and schooling. All the participants underwent a clinical interview, tone audiometry, otorhinolaryngological evaluation and cognitive assessment of phonological working memory (numbers and pseudowords), reading comprehension and arithmetic skills.

**RESULTS:**

Children with mouth breathing had poorer performance than controls, regarding reading comprehension (P = 0.006), arithmetic (P = 0.025) and working memory for pseudowords (P = 0.002), but not for numbers (P = 0.76).

**CONCLUSION:**

Children with mouth breathing have low academic achievement and poorer phonological working memory than controls. Teachers and healthcare professionals should be aware of the association of mouth breathing with children’s physical and cognitive health.

## RESUMO

**CONTEXTO E OBJETIVO:**

A síndrome da respiração oral é muito comum em crianças em idade escolar, e está possivelmente relacionada a dificuldades de aprendizagem e baixo rendimento escolar. Neste estudo, investigamos memória operacional, compreensão de leitura e habilidades aritméticas em crianças com respiração nasal e oral.

**TIPO DE ESTUDO E LOCAL:**

Estudo transversal analítico com grupo controle realizado em hospital universitário público.

**MÉTODOS:**

42 crianças (média = 8,7 anos) identificadas com respiração oral foram comparadas a um grupo controle (média = 8,4 anos) e pareadas por idade e escolaridade. Todos os participantes foram submetidos a entrevista clínica, audiometria tonal, avaliação otorrinolaringológica e avaliação cognitiva da memória operacional fonológica (números e pseudopalavras), compreensão de leitura e aritmética.

**RESULTADOS:**

Crianças com respiração oral tiveram desempenho significativamente inferior ao de respiradores nasais em compreensão de leitura (P = 0,006), aritmética (P = 0,025) e memória operacional para pseudopalavras (P = 0,002), mas não para números (P = 0,76).

**CONCLUSÕES:**

Crianças com respiração oral apresentam baixo rendimento escolar e menor memória operacional fonológica em comparação ao grupo controle. Professores e profissionais da saúde devem atentar para a associação da respiração oral com a saúde física e cognitiva das crianças.

## INTRODUCTION

Mouth breathing syndrome is a very common condition that affects around 50% of Brazilian children at school age.^1^^,^^2^ It is characterized by nasal obstruction symptoms (e.g. hyponasal speech, sleep fragmentation, snoring during sleep and drooling) and a set of body adaptations, such as forward head posture and low forward tongue position.^3^^,^^4^ The main causes are allergic rhinitis, palatine and/or pharyngeal tonsil hypertrophy, and septal deviation.^3^^,^^4^

Chronic mouth breathing may have several consequences, such as dentofacial morphological and orofacial myofunctional adaptations,^5^^,^^6^^,^^7^ along with changes to body posture,^3^^,^^4^^,^^8^^,^^9^^,^^10^^,^^11^ pulmonary function,^10^ auditory processing and voice.^12^^,^^13^^,^^14^^,^^15^ In some cases, mouth breathing is associated with sleep-related obstructive breathing disorders, with important behavioral and cognitive impacts such as daytime tiredness, sleepiness, poor concentration and attention, and such children may often present learning difficulties and low academic achievement.^16^^,^^17^^,^^18^

However, little is known about working memory problems in children with mouth breathing. Working memory is the cognitive system responsible for temporary storage and processing of information during complex cognitive tasks, and it is crucially involved in reading comprehension, arithmetic skills and academic achievement.^19^^,^^20^^,^^21^^,^^22^ Hence, there are strong reasons to expect that children with mouth breathing may also have working memory problems. In the present study, children with mouth breathing were compared with a control group in relation to a series of cognitive tasks such as reading comprehension, arithmetic and phonological working memory.

## OBJECTIVE

The objective of this study was to investigate the cognitive and academic skills of children with mouth breathing.

## METHODS

### Ethics

This study was approved by the Research Ethics Committee of the university hospital (Process HCRP-9503/2007). The directors and coordinators of two public schools were contacted and they authorized selection of children. In addition, the children’s parents or guardians received information about the study and signed a consent form.

### Design and sample size

This was an analytical cross-sectional study with a control group. The sample size was calculated based on a pilot study consisting of the first six participants in each group. Taking an alpha of 0.05, power (1 - beta) of 0.95 and effect size (d) of 1.33 (as derived from the pilot study), it was estimated that at least 13 participants per group would be required in order to detect significant differences between the groups.

### Participants

For this study, we recruited children aged between 7 and 10 years (i.e. an age range from 84 to 120 months), who were students in the second or third year of public elementary schools. Eligible participants were recruited among the children who were attending the Mouth Breathing Center of the university hospital. Children and their parents who were considered eligible according to the inclusion criteria were simply invited to participate, and 31 families agreed. The 31 children attended a complete otorhinolaryngological evaluation.

Participants for the control group were recruited from two local public elementary schools, and were also students from the second and third years. Forty invited children whose parents had signed the consent form were scheduled to attend a complete otorhinolaryngological evaluation, but only 24 actually went to the university hospital. During the evaluation, 13 children were assigned to the control group and 11 children were identified as presenting mouth breathing, thus giving a total of 42 children in the mouth breathing group.

The participants had to meet specific criteria to enter the study. Firstly, during the anamnesis, the parents or guardians answered questions regarding the child’s clinical history and health, including physical, emotional, behavioral and socioenvironmental factors. Children reported as having any psychological or neurological problems, as well as children undergoing pedagogical or speech-therapy interventions, were not included in the sample. Secondly, the child underwent tone audiometry in a soundproof cabin. Air-conduction thresholds were measured in both descending format (10 dB intervals) and ascending format (5 dB intervals) at frequencies of 250, 500, 1000, 2000, 3000, 4000, 6000 and 8000 Hz. Children with any degree of hearing loss were not included in the sample, i.e. children whose average thresholds for all the frequencies were greater than 15 dB.

Finally, the children underwent otorhinolaryngological evaluation. A clinical questionnaire assessed both the intensity and the frequency of obstructive symptoms. Anterior rhinoscopy was performed to investigate color (pale, ruddy or hyperemic) and trophism (normotrophic, hypotrophic or hypertrophic) of the inferior turbinates, as well as the presence of septal deviation. Oroscopy was used to investigate the level of palatine tonsil hypertrophy, according to the classification of Brodsky and Koch.^23^ Nasoendoscopy was used to investigate the nasal structures and percentage of adenoid tissue in the nasopharynx. If the pharyngeal tonsil occupied more than 70%, it was considered to be obstructive: this is the standard parameter used in our service.

The mouth breathing group included children with one or more of the following characteristics: (1) obstructive or irritating nasal signs lasting more than three months; (2) obstructive tonsils (palatine tonsil degrees III or IV, and/or pharyngeal tonsil occupying more than 70% of the nasopharynx); (3) inferior turbinate hypertrophy; and (4) obstructive septal deviation.

The control group included children with the following characteristics: (1) obstructive or irritating nasal signs occurring a maximum of four times a year, with each episode lasting less than 15 days; (2) non-obstructive palatine tonsil (palatine tonsil degrees I or II, and pharyngeal tonsil occupying less than 70% of the nasopharynx); (3) normotrophic inferior turbinate; and (4) no obstructive septal deviation.

### Materials and procedure

The participants were individually tested in a silent room. The cognitive assessment included the tests described below.

Reading comprehension: The Sentence Reading Competency Test (TCLS 1.1), designed by Capovilla et al.^24^ was used. This is a brochure containing five practice trials and 40 test trials. Each trial consisted of a sentence followed by five pictures among which a single picture expressed the meaning of the sentence. The length of the sentences and their lexical and syntactical complexity increased over the course of the test. The children scored one point for each correct trial (maximum raw score = 40 points).

Arithmetic: The arithmetic subtest from the School Performance Test (Teste de Desempenho Escolar, TDE), an academic achievement test proposed by Stein,^25^ was used. The TDE is a test widely used in Brazil to assess academic achievement between the first and sixth years of elementary school. In particular, the arithmetic subtest comprises two parts: an oral part that involves solving three problems, and a written part containing 35 arithmetic operations of increasing level of difficulty (6 addition tasks, 8 subtraction tasks, 4 multiplication tasks, 5 division tasks, 7 exercises involving fractions, 2 exponentiation tasks and 3 numerical expressions). The children scored one point for each correct answer (maximum raw score = 38), and it should be noted that children around 8.5 years of age are expected to have an average score of around 10-13 points.

Phonological working memory (number repetition): The Auditory Sequential Memory subtest 5 from the Illinois Test of Pschycholinguistic Abilities (ITPA-5), which was adapted and validated for the Brazilian population by Bogossian and Santos,^26^ was used. The child had to repeat number sequences of increasing length, containing from two to seven items. Two attempts at each sequence were allowed in case of mistakes at the first attempt. Children scored two points for each correct sequence at the first attempt, and one point for a successful second try. There was a total of 21 sequences in the test, and administration of the test was halted if the sequences of a given length were not fully recalled at both attempts (maximum raw score = 42 points).

Phonological Working Memory (pseudoword repetition): The pseudoword repetition test of Kessler^27^ was used. The child had to repeat a list of five pseudowords, and there were six lists of increasing complexity: the first list had five monosyllabic pseudowords, the second list had five disyllabic items, and so on. The child scored one point for each item recalled correctly (maximum raw score = 30).

### Statistical analysis

For each task, the nonparametric Mann-Whitney U test was used to compare raw scores from two independent samples (children with mouth breathing syndrome and controls), with the significance level α = 0.05 and *r* as the effect-size indicator.

## RESULTS

The sample comprised 55 children divided into two groups according to the otorhinolaryngological evaluation: 42 children were identified as presenting mouth breathing (MB group) and 13 children, nasal breathing (control group). Table 1 summarizes the demographic information about the sample.

**Table 1.**
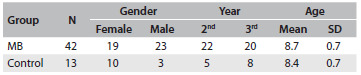
Demographic characteristics of participants entering the study

MB = mouth breathing; SD = standard deviation.

The results from the otorhinolaryngological evaluation revealed that the majority of mouth-breathers have turbinate hypertrophy (85%), bilateral palatine tonsil hypertrophy degrees III (39.5%) or IV (10.5%), pharyngeal tonsil occupying more than 70% of the nasopharynx (32.5%) and septal deviation (16.2%). The results from the anterior rhinoscopy, oroscopy and nasoendoscopy are summarized in Tables 2, 3 and 4, respectively.

**Table 2.**
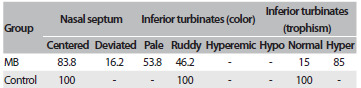
Results from anterior rhinoscopy: percentage of participants in each classification of nasal septum and inferior turbinates (color and trophism)

MB = mouth breathing.

**Table 3.**
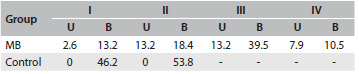
Results from oroscopy: percentage of participants observed at each level of palatine tonsil hypertrophy (levels I to IV), with either unilateral (U) or bilateral (B) involvement

MB = mouth breathing.

**Table 4.**
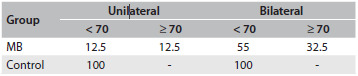
Results from nasoendoscopy: percentage of participants according to amount of adenoid tissue in the nasopharynx

MB = mouth breathing.

The results from the cognitive assessment for each group of participants are summarized in Table 5. Control children were better than children with MB in the reading comprehension test (U = 137.5, Z = 2.69, P = 0.006, r = 0.36) and in the arithmetic test (U = 160.5, Z = 2.24, P = 0.025, r = 0.30). No significant differences between the groups were observed in relation to phonological working memory for numbers (U = 257, Z = 0.32, P = 0.76, r = 0.04) or pseudowords (U = 178, Z = 1.88, P = 0.06, r = 0.25). However, in this latter case, it should be noted that both the P-value and the effect size (r) suggest that was a discrepancy between the groups.

**Table 5.**
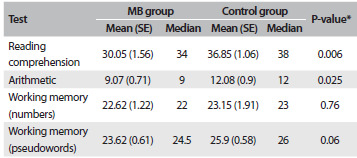
Mean scores, standard errors (SE) and medians for the two groups of participants in each task

MB = mouth breathing; ^*^Mann-Whitney U test.

**Figure 1.**
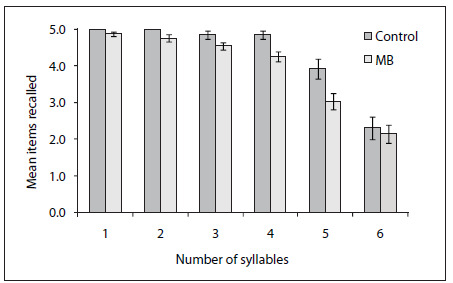
Mean number of pseudowords recalled by the control and mouth breathing (MB) groups as a function of the number of syllables. Error bars represent standard errors of the mean.

In order to explore the differences between the groups regarding pseudoword repetition, we separated the participants’ performance for each level of complexity, i.e. from one to six syllables. As shown in Figure 1, discrepancies between the groups emerged with increasing numbers of syllables, whereas a drop in performance for the longest list (five items with six syllables each) was observed for both groups. This drew our attention to the plausibility and validity of this test, given that very long words are not frequent in Portuguese. By excluding level six from the scoring, a highly significant difference between the groups was observed (U = 122.5, Z = 3.01, P = 0.002, r = 0.41).

## DISCUSSION

This study evaluated cognitive skills relating to reading comprehension, arithmetic and phonological working memory among children with mouth breathing syndrome and control children with typical nasal breathing.

With regard to the sample, it should be noted that the inclusion criteria allowed different profiles of mouth breathers to be selected. In fact, mouth breathing is a multifactorial syndrome and our primary concern was to reflect this heterogeneity. Interestingly, our sample seems to have reflected the prevalence data described in the literature,^1^^,^^2^ i.e. the main causes of mouth breathing: allergic rhinitis, palatine and/or pharyngeal tonsil hypertrophy, and septal deviation. As observed, the predominant obstruction was turbinate hypertrophy, which is often associated with allergic rhinitis.^3^^,^^4^ However, instead of focusing on specific categories of diagnosis, we used the broader category of mouth breathing. The motivation for this study was the observation that children who attend the Mouth Breathing Center of the university hospital often present learning difficulties and low school achievement.

With regard to the cognitive assessment, the results are clear-cut: the children with mouth breathing had poorer academic achievement and cognitive skills than the control children. In the reading comprehension test, the control children were able to accurately select target pictures, thereby indicating satisfactory linguistic skills and language comprehension. On the contrary, children with mouth breathing selected more distracting pictures, thus suggesting difficulties in dealing with syntactical complexity and in understanding written language. The MB group also had lower scores than the control group in the arithmetic test, thereby indicating difficulties with numerical operations. These results further support the association of mouth breathing with poor learning and school performance.^16^^,^^17^^,^^18^ In general, mouth breathing is assumed to impair children’s overall health through causing daytime tiredness and loss of attention. Given that reading and calculating depend on temporary processing and retention of information, we also expected differences in children’s working memory.

With regard to phonological working memory, the differences between the groups were less evident. The two groups had similar performance in the number subtest of the ITPA-5, whereas the control children tended to recall more pseudowords than the mouth-breathers. This tendency was observed in the children’s overall performance in the test, which takes into consideration the number of items recalled correctly at all six levels of complexity. However, a clear difference between the groups was observed after excluding the list with six syllable pseudowords. This result suggests that the test lost its discriminatory power with long pseudowords, which instead should reflect the typical syllabic pattern of Portuguese. In general, pseudoword repetition seems to be appropriate for assessing phonological working memory, given that the participants have to rely on temporary retention of phonological information. On the other hand, numbers are very simple and frequent items, and number repetition may not be appropriate for detecting differences between the groups in relation to working memory.

In summary, our results suggest that mouth breathing may be linked to poor academic achievement and phonological working memory, thus providing additional evidence that breathing pattern problems have negative impacts on attention and memory, and lead to poor motivation and learning.^16^^,^^17^^,^^18^ However, only an extensive evaluation of children’s skills would provide detailed information about the cognitive impairments relating to mouth breathing. Future research should use a wider range of tests to assess language learning, vocabulary knowledge and verbal reasoning, as well as executive functions^20^^,^^21^^,^^22^ (e.g. inhibition, divided attention and task switching) and other working memory domains (e.g. visuospatial working memory). Importantly, future research should also investigate whether clinical and/or surgical interventions are effective for reducing cognitive impairments and enhancing learning and school performance.

## CONCLUSIONS

The present study indicates that mouth breathing may be linked to poor school performance, resulting from impairments in reading comprehension, arithmetic and working memory. Given that mouth breathing is very common in children at school age, teachers and healthcare professionals should be aware of its negative impacts on children’s physical and cognitive health.
